# Associations Between a New Biomarker of Elevated Chronic Inflammation and Accelerated Aging

**DOI:** 10.1093/geroni/igaa057.464

**Published:** 2020-12-16

**Authors:** Line Rasmussen, Avshalom Caspi, Terrie Moffitt

**Affiliations:** Duke University, Durham, North Carolina, United States

## Abstract

To further understand and measure the association between chronic inflammation, aging, and age-related diseases, broadly applicable standard biomarkers of systemic chronic inflammation are needed. We tested whether elevated blood levels of the emerging chronic inflammation marker soluble urokinase plasminogen activator receptor (suPAR) were associated with accelerated aging, lower functional capacity, and cognitive decline. We used data from the population-representative longitudinal Dunedin Study (N=875). Plasma suPAR levels were analyzed at ages 38 and 45 years. We performed regression analyses adjusted for sex, smoking, and C-reactive protein. suPAR levels increased from 2.39 ng/mL (SD 0.89) at age 38 to 3.01 (SD 1.03) at age 45 years. Elevated suPAR was associated with accelerated pace of biological aging across multiple organ systems (β 0.28, 95% CI 0.21–0.35), older facial appearance (β 0.16, 95% CI 0.10–0.22), and with structural signs of older brain age (β 0.06, 95% CI -0.00–0.13). Moreover, participants with higher suPAR levels had lower functional capacity (more physical limitations [β 0.24, 95% CI 0.18–0.30]; slower gait speed [β -0.14, 95% CI -0.20; -0.08]) and greater decline in cognitive function (β -0.07, 95% CI -0.13; -0.01) from childhood to adulthood compared to those with lower suPAR levels. Finally, improvements in health habits between age 38 and 45 (smoking cessation or increased physical activity) were associated with less steep increases in suPAR levels over those years. Our findings provide initial support for the utility of suPAR in studying the role of chronic inflammation in accelerated aging and functional decline.

